# A 3D-Printable Polymer-Metal Soft-Magnetic Functional Composite—Development and Characterization

**DOI:** 10.3390/ma11020189

**Published:** 2018-01-25

**Authors:** Bilal Khatri, Karl Lappe, Dorit Noetzel, Kilian Pursche, Thomas Hanemann

**Affiliations:** 1 Laboratory for Materials Processing, University of Freiburg, 79110 Freiburg, Germany; lappe@posteo.de (K.L.); thomas.hanemann@kit.edu (T.H.); 2 Karlsruhe Institute of Technology, Institute of Applied Materials, 76344 Karlsruhe, Germany; dorit.noetzel@kit.edu (D.N.); kilian.pursche@kit.edu (K.P.)

**Keywords:** polymer–metal composite, 3D printing, fused deposition modeling, material characterization, mechanical characterization, magnetic characterization

## Abstract

In this work, a 3D printed polymer–metal soft-magnetic composite was developed and characterized for its material, structural, and functional properties. The material comprises acrylonitrile butadiene styrene (ABS) as the polymer matrix, with up to 40 vol. % stainless steel micropowder as the filler. The composites were rheologically analyzed and 3D printed into tensile and flexural test specimens using a commercial desktop 3D printer. Mechanical characterization revealed a linearly decreasing trend of the ultimate tensile strength (UTS) and a sharp decrease in Young’s modulus with increasing filler content. Four-point bending analysis showed a decrease of up to 70% in the flexural strength of the composite and up to a two-factor increase in the secant modulus of elasticity. Magnetic hysteresis characterization revealed retentivities of up to 15.6 mT and coercive forces of up to 4.31 kA/m at an applied magnetic field of 485 kA/m. The composite shows promise as a material for the additive manufacturing of passive magnetic sensors and/or actuators.

## 1. Introduction

Additive manufacturing (AM) or rapid prototyping (RP)—more commonly known as 3D printing—has gained a great deal of traction over the past two decades as a family of versatile manufacturing techniques, even being deemed “world-changing” [1]. In contrast to traditional manufacturing methods, AM allows for the development of macroscopic and microscopic parts and structures built from the ground up. Recent publications and reviews of different AM technologies [2,3] show applications as diverse as casting [4,5], MEMS [6,7,8], micro and embedded optics [9,10,11], micro-fluidics [12,13], biomaterials and biotechnology [14,15,16,17,18,19,20,21,22], and food manufacturing [23,24].

The role of composite materials has been crucial for the success and propagation of AM technologies, owing to their useful functional properties being combined with the flexibility of 3D printing. Fused deposition modeling (FDM, first coined by Stratasys®) or fused filament fabrication (FFF) is the one of the most ubiquitous AM techniques today, involving the layer-by-layer deposition of melted thermoplasts on to a build-platform. In recent years, the focus has shifted towards the development of 3D-printable functional composites [25,26] with the vision of assisting (or in some cases, replacing) traditional manufacturing technologies. Numerous investigations of composite materials for FDM have been undertaken, involving filler materials such as glass [27], ceramics [28,29,30,31], and metals [32,33]; characterized for their thermomechanical [34,35,36,37,38,39], electric [40,41,42], dielectric [43,44], and ferromagnetic [45] properties. Other studies have looked at the magnetic properties of polymer matrix composites, including those with Ni [46], ferrite [47,48], and Nd-Fe-B [49] as functional materials. However, these did not look at AM as a forming method.

In this study, a 3D-printable soft-magnetic composite material is presented, comprising a well-established polymer in acrylonitrile butadiene styrene (ABS) as the matrix material, filled with stainless steel microparticles (17-4PH)—a known soft-magnetic material used in micro injection molding [50]. A recent study [51] investigated 17-4PH powder for AM using selective laser sintering (SLS). The stainless steel powder was chosen for its soft magnetic properties, leading to a potential use in the additive production of magnetic–nonmagnetic bimetals [52] and in magnetic sensing applications [53,54]. At high filler ratios, a sintering step can lead to pure metallic structures [55].

The development process begins with the analysis of the filler particles, through the development of the composite feedstocks and filaments in four different filler ratios, to their material, optical, and rheological characterization. A commercially available 3D printer was used to print test samples, which could then be characterized for their structural and functional properties. These properties were then correlated back to the properties of the pure polymer, thus revealing the influence of the filler material. The results show that the composite acts as a soft-magnetic material, reliably printable at filler ratios of up to 40 vol. %, and provide a strong basis for the development of additively manufactured magnetic sensors or magnetic–nonmagnetic bistrips.

## 2. Materials and Sample Preparation

The polymer–magnetic composite comprised acrylonitrile butadiene styrene (ABS) pellets (Terluran® GP-22, Styrolution, Frankfurt, Germany) as the matrix material, and stainless steel micropowder Osprey 17-4PH (Sandvik-Osprey, Neath, UK) as the filler. Four different composites were prepared, in volumetric ratios of 10, 20, 30, and 40 vol. %. A feedstock with 50 vol. % filler was also prepared, but it was difficult to work with due to its high viscosity and brittle characteristics.

### 2.1. Composite Preparation

**Figure 1:**
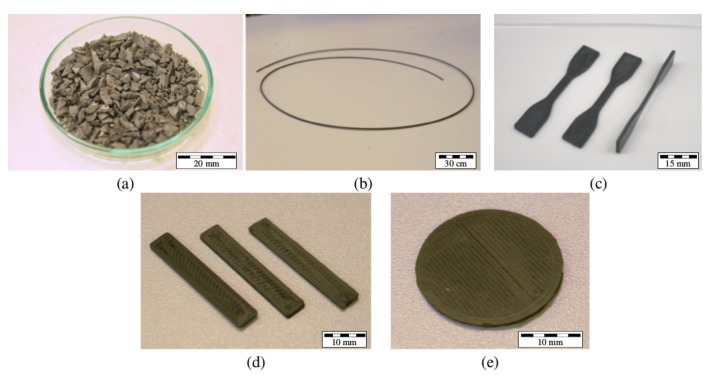
(**a**) The acrylonitrile butadiene styrene (ABS)–Steel composite feedstock after kneading and drying. (**b**) The extruded composite filament. (**c**) Tensile test samples. (**d**) Flexural test specimens. (**e**) A sample for magnetic characterization.

The material composites were prepared by heated kneading of the ABS pellets with the steel micropowder in a Brabender W50-EHT (Brabender GmBH, Duisburg, Germany) kneader for 60 min at 180 °C. The composite feedstocks were dried in an oven for 24 h at 130 °C to minimize moisture, as it was found to adversely affect the extrusion process due to the formation of voids. The composite feedstock can be seen in Figure 1a.

The feedstocks were extruded into filaments using the Noztek Pro single barrel extruder (Noztek, Shoreham, England) at temperatures between 190 °C and 210 °C. Pure ABS filaments were also extruded at 190 °C for comparison. The target filament diameter of 1.75 mm ±10 mm was achieved by adjusting the spooling speed of the extruder.

This target diameter was not consistently achievable for the feedstock with 50 vol. % feedstock due to its high viscosity and phase separation between the ABS and steel particles. Moreover, the filament samples prepared using this feedstock were too brittle to be reliably printed, as they would fail inside the filament-feed drive of the 3D printer.

### 2.2. Material Characterization

The steel micropowder was deagglomerated using ultrasound and characterized for its particle size using the Beckman Coulter LS-230 particle counter (Beckman-Coulter Inc., Brea, CA, USA) in an isopropanol medium.

The composite feedstocks were rheologically characterized in the Goettfert Rheograph 25 capillary rheometer (Goettfert GmBH, Buchen, Germany) starting at a shear rate of 2 s^−1^ up to at least 300 s^−1^ for all composites at 180 °C. Higher shear rates were measured for composites with lower filler content.

Thermogravimetry was carried out for pure and composite feedstock, filaments, and printed test samples using the Netzsch STA-409 differential calorimeter (Netzch Group, Selb, Germany) in an inert (argon) atmosphere. The samples were heated to 1000 °C at 10 K/min and held for 30 min.

Microscope images of the test samples were taken with the Zeiss Axioplan 2 (Carl Zeiss Microscopy GmBH, Jena, Germany) at 20× and 50× magnification.

### 2.3. Sample Printing

All test samples were printed using the MakerBot Replicator 2X (MakerBot, New York, NY, USA) desktop 3D printer. The tensile samples were printed in accordance with the ASTM D638 (Type IV) standard with the average dimensions 57 (*l*) × 3.5 (*b, centre*) × 1.5 (*h*) mm^3^. The flexural specimens were printed based on the ASTM D7264M standard at 30 (*l*) × 5.5 (*b*) × 1.5 (*h*) mm^3^. The samples for magnetic characterization were printed as discs using the DIN EN 60404-5 standard with a diameter of 25 mm and a height of 2 mm. Pure ABS samples were printed at 230 °C and all composite samples were structured at 240 °C. All samples were printed in a laid-down orientation, such that each layer within a specimen was identical (Figure 1c–e). Table 1 lists the print parameters for the test samples.

### 2.4. Structural Characterization

Tensile and bending tests were performed using the Zwick Z010 universal testing machine (Zwick/Roell, Ulm, Germany). Five samples were tested for each composite variant. Tensile tests were carried out at 5 mm/min at a maximum load of 5 kN. The four-point flexural tests were made with the loading span at one-third of the support span at a speed of 2mm/min.

### 2.5. Functional Characterization

Magnetic BH analysis was performed on the Steingroever Permagraph C-500 (Steingrover GmBH, Cologne, Germany). The maximum applied magnetic field was 485 kA/m to ensure magnetic saturation. The hysteresis curve, including the magnetic retentivity and coercive force, were measured for each composite.

## 3. Results and Discussion

### 3.1. Material Characterization

#### 3.1.1. Particle Size and Distribution

**Figure 2:**
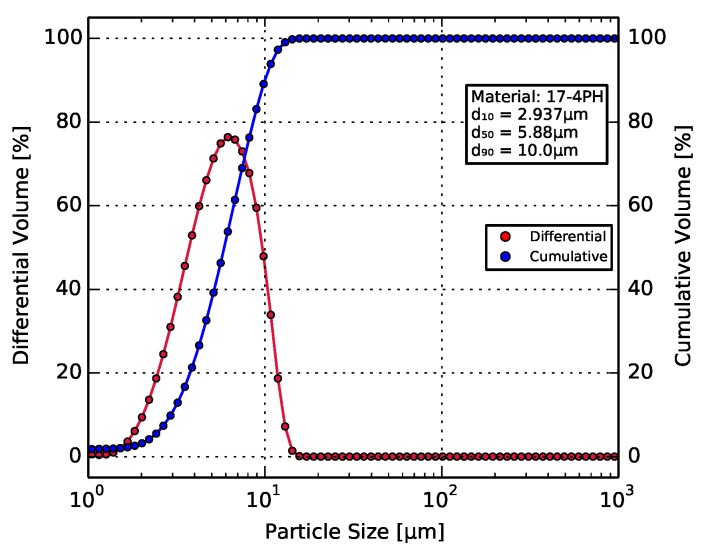
Particle size distribution of the steel micropowder, with a *d*_50_ value of 5.88 μm.

The steel micropowder exhibited a monomodal particle size distribution (as shown in Figure 2), with a *d*_50_ lying at 5.88 μm. This agreed well with the manufacturer-provided values.

**Figure 3:**
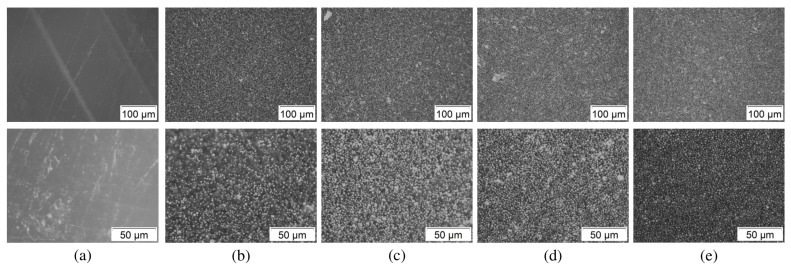
Microscopy images test sample cross-sections at (**top**) 20× and (**bottom**) 50× zoom. (**a**) Pure ABS, (**b**) 10 vol. %, (**c**) 20 vol. %, (**d**) 30 vol. %; and (**e**) 40 vol. %.

Extruded filaments and test sample cross-sections were analyzed for the steel particle distribution in the polymer matrix. Figure 3 shows specimen cross-section microscopy images at 20× and 50× zoom. The white regions indicate the steel particles held in the matrix. The filaments as well as the sample cross-sections exhibited a uniform particle distribution. Minimal localized non-uniformities could be seen due to the presence of agglomerates or air pockets. These remained consistent throughout the processing and printing steps, and could be improved upon through longer mixing times.

#### 3.1.2. Rheology

**Figure 4:**
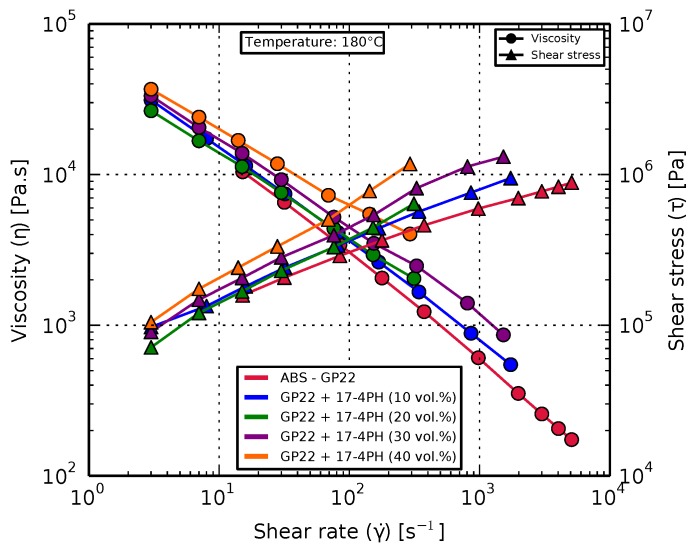
Rheological behavior of pure ABS compared with the composites exhibiting a shear-thinning behavior. Increase in filler content led to increased viscosity for a given shear rate.

Feedstock samples of pure ABS and the four composite variants were characterized for their viscosity and shear stress. All variants exhibited the expected non-Newtonian shear-thinning behavior [56], with the increase in filler content increasing the viscosity, as shown in Figure 4. Table 2 lists the viscosity and shear stress values for shear rates between 200 s^−1^ and 300 s^−1^.

A feedstock with 50 vol. % filler was additionally prepared (see Section 2.1) and tested. Its viscosity was seen to be beyond the measurement range of the rheometer, reaching a value of 100 kPa at shear rates as low as 70 s^−1^.

#### 3.1.3. Thermogravimetry

**Figure 5:**
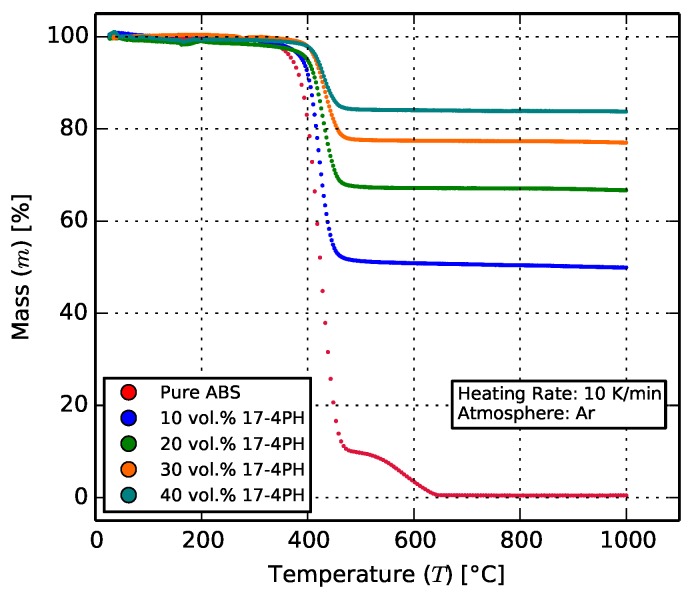
Thermogravimetric behavior of the composites. The organic ABS burns away between 400 °C and 600 °C, leaving behind the metallic content of the composite. For the 40 vol. % composite, this translates to 83.7 wt. %.

Thermogravimetric analysis was performed in an inert Ar atmosphere to prevent oxidation of the metals comprising the steel filler. Figure 5 shows the results obtained. Pure ABS burned away between 400 °C and 600 °C, leaving behind the filler, which was measured by weight. The remaining filler content for each composite as a percentage of the total sample mass is listed in Table 3.

The 50 vol. % feedstock (see Section 2.1) had a residual mass of 89.0 wt. %. This composite could not be reliably extruded or 3D printed, and as such is omitted from the results that follow.

The results from thermogravimetric analysis remained consistent for the feedstock and filament, thereby ensuring minimal influence of the processing steps on the matrix-to-filler ratio.

### 3.2. Structural Characterization

#### 3.2.1. Tensile

**Figure 6:**
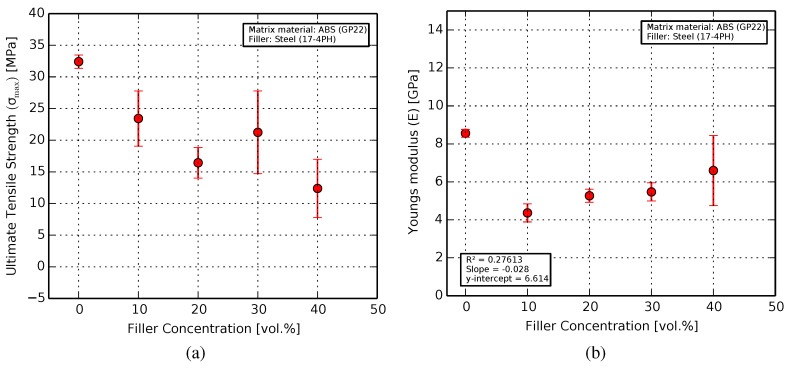
Results from the tensile characterization showing (**a**) the ultimate tensile strength decreasing by up to 61% compared to pure ABS; and (**b**) the Young’s modulus decreasing by about 50% for the 10 vol. % samples.

Tensile characterization of the samples revealed an exceedingly brittle characteristic with increasing filler content. The ultimate tensile strength of the printed samples decreased from 32.4 MPa for pure ABS (which agrees well with the literature [57,58]), to 12.5 MPa for the 40 vol. % composite (Figure 6a). This decrease is due to the presence and concentration of the comparatively brittle filler particles in the matrix.

The Young’s modulus showed a decrease of about 50% from pure ABS (at 8.48 MPa) [59] to the 10 vol. % composite (at 4.2 MPa) as seen in Figure 6b. Further increase in filler content, however, showed a slight rise in the mean values of Young’s modulus, with the 20 vol. % composite at 5.2 GPa and the 40 vol. % at 6.5 GPa. Taking the error bars into account, the Young’s modulus can be considered constant for this range.

The steel micro-particles in spherical form act as defects in the polymer matrix, leading to a marked decrease in bulk ductility, and consequently the ultimate tensile strength (UTS) and Young’s modulus (Figure 6) with increasing filler content. The relatively large standard deviation in the measurements can be attributed to agglomerates and clumps of the filler as well as air pockets inside the test samples, resulting in premature failure.

Conversely, regions with a lack of filler particles resulted in greater plastic deformation before failure. This effect was most pronounced for the 10 vol. % samples with unusually large plastic regimes and strains of up to 12%, resulting in the low Young’s modulus.

The spatial distribution of these inhomogeneous regions also adversely affect the sample quality. Homogeneous particle distribution in the polymer through longer kneading/mixing times, the use of a deagglomerizing agent, and larger sample sizes for each point can improve these results.

#### 3.2.2. Flexural

**Figure 7:**
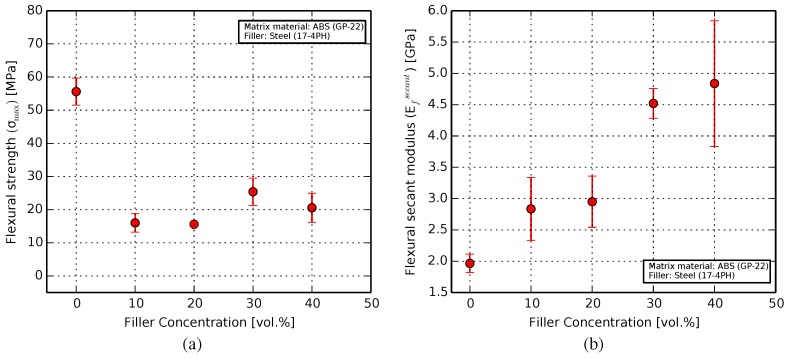
Results from the flexural characterization of pure ABS and composite samples: (**a**) Flexural strength showing a sharp decrease in flexibility due to the inclusion of filler particles; (**b**) The flexural secant modulus of elasticity increasing by over a factor of two for the composites, compared to pure ABS.

The flexural strength was seen to decrease by 72%, from an expected 55.6 MPa [60] for pure ABS to between 15.5 MPa and 25.4 MPa for the composites, as seen in Figure 7a.

The secant modulus of elasticity (Figure 7b) was measured between the starting position and one-third of the maximum deflection to ensure values within the linear region of deformation. It saw an increase from 1.96 GPa for pure ABS [36] to 4.83 GPa for the 40 vol. % sample.

The flexural strength of the composites was up to 72% lower than that of pure ABS. This is in part due to the inclusion of the fillers. However, the print orientation plays a significant role here. The bending force on the samples acts at a direction perpendicular to that of the print-layers. In this configuration, the sample failure depends highly on the proximity of the inhomogeneous or locally brittle areas along the length of a given sample to the points where the force is applied. This effect is seen to dominate the intrinsic flexibility of the composite probes, leading to a fairly similar result for all composites.

The secant module of elasticity was calculated in the linear region of the force-deflection curve, and thus shows the intrinsic flexural modulus of the samples, without the adverse effect of the experiment on the bulk ductility. Here, the secant modulus can be seen to increase with filler ratio, owing to the higher resistance offered by the steel particles. Based on tensile testing performed earlier, this increase was predicted to be linear. The closeness of the 10 and 20% samples to each other, as well as that of the 30 and 40% specimens, can be improved upon by the use of larger sample sizes.

### 3.3. Functional Characterization

**Figure 8:**
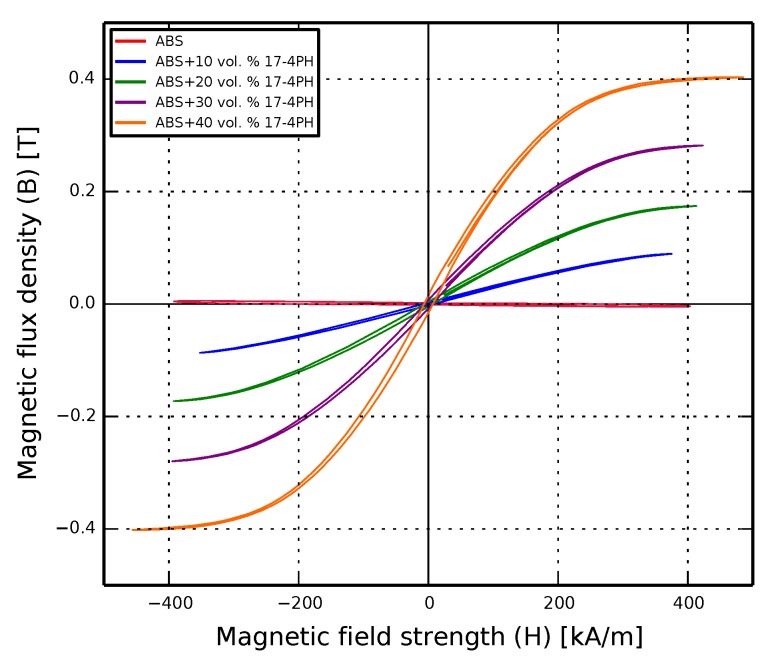
Results from the magnetic characterization of the composites, showing a maximum retentivity of 15.6 mT for the 40 vol. % sample.

Functional characterization of the composites showed the expected increase in ferromagnetism at higher filler ratios. Pure ABS had no significant magnetic response. The samples with 40 vol. % steel exhibited a magnetic retentivity of 15.6 mT at an applied field of 485 kA/m, as illustrated in Figure 8. Table 4 shows the numerical results.

The results show a soft-magnetic response of the composite, with the retentivity (*B_T_*) increasing by around a factor of two for every 10 vol. % increase in filler ratio. The samples were given no post-print treatment, and even so exhibit characteristics comparable to those of studies with other composites which used traditional sample preparation methods [46,47]. The doubling of magnetic retentivity with filler content is promising, as at higher filler ratios, the composite is a candidate for the rapid manufacturing of structures for magnetic sensing applications.

## 4. Conclusions

In this work, 3D-printable polymer–metal soft-magnetic functional composites were developed in filler ratios of up to 40 vol. %. The composite feedstocks were analyzed for their material attributes, and were 3D printed and characterized for their structural and functional properties. The results at each development step exhibited repeatable results, such as the filler-particle content and their distribution in the polymer matrix. The inclusion of filler particles showed a pronounced decrease in the structural strength when compared to that of the pure polymer, and an increase in the functional magnetic response. At higher filler ratios, the composite is expected to exhibit stronger magnetic properties. The combination of mechanical and magnetic properties can be applied in a system which contains integrated 3D-printed magnetic volumes or structures that experience mechanical loads.

These results provide a solid proof-of-concept and can be useful as a basis for the additive manufacturing of bimetals in magnetic sensing applications. The composite shows promise as a functional magnetic material which can be manufactured with high structural freedom using a commercially available 3D printer, hindered only by the composite filament being too brittle for the printer at high filler ratios. This challenge can be overcome by modifying the printer to use softer materials in the printer’s filament feed mechanism, by the use of a more ductile polymer as the matrix material, or by the inclusion of additives that increase the bulk ductility of the composite.

For improved magnetic responses, a different filler material could also be investigated by following the procedure described in this work. The characterization results as such provide a basis upon which future studies could be built, leading to the development of structures with predictable and reliable mechanical and functional properties.

## Figures and Tables

**Table 1:** Parameters used for the 3D printing of the test samples.

Layer Thickness (mm)	Infill (%)	Build-Platform Temperature (°C)	Number of Shells	Raster Angle (°)
0.2	100	110	3	45

**Table 2:** Rheological behavior of pure ABS and composite feedstocks.

Filler Ratio (vol. %)	Shear Rate (s^−1^)	Shear Stress (MPa)	Viscosity (kPa s)
0	374	4.60	1.22
10	342	5.67	1.66
20	313	6.39	2.04
30	328	8.12	2.47
40	292	11.75	4.02

**Table 3:** The residual masses for each of the composites after thermogravimetry.

Filler Ratio (vol. %)	Remaining Mass (%)
0	0.47
10	49.9
20	66.7
30	77.0
40	83.75

**Table 4:** Results from the magnetic characterization.

Filler Ratio (vol. %)	Max. Field Strength	Max. Flux Density	Retentivity	Coercive Force	Coercive Force
	(*H_max_*)	(*B_max_*)	(*B_r_*)	(*H_cB_*)(−ve)	(*H_cJ_*)(+ve)
	(kA/m)	(mT)	(mT)	(kA/m)	(kA/m)
0	404	2.57	1.79	1.51	226
10	375	89.2	2.31	1.49	7.72
20	413	174	4.15	2.15	6.05
30	423	282	7.11	2.83	5.62
40	485	403	15.6	4.31	6.51

## Abbreviations

The following abbreviations are used in this manuscript:

AM	Additive manufacturing
RP	Rapid prototyping
FDM	Fused deposition modeling
FFF	Fused filament fabrication
ABS	acrylonitrile butadiene styrene
SLS	Selective laser sintering
UTS	ultimate tensile strength
